# Predictive modeling and regression analysis of diverse sulfonamide compounds employed in cancer therapy

**DOI:** 10.3389/fchem.2024.1413850

**Published:** 2024-05-27

**Authors:** Muhammad Danish, Tehreem Liaquat, Farwa Ashraf, Shahid Zaman

**Affiliations:** ^1^ Department of Chemistry, University of Sialkot, Sialkot, Pakistan; ^2^ Department of Mathematics, University of Sialkot, Sialkot, Pakistan

**Keywords:** compounds with anti-cancer/anti-tumor sulfonamides, topological indices(TIs), quantitative structure-property relationship QSPR analysis, and regression models, graph theory

## Abstract

Topological indices (TIs) have rich applications in various biological contexts, particularly in therapeutic strategies for cancer. Predicting the performance of compounds in the treatment of cancer is one such application, wherein TIs offer insights into the molecular structures and related properties of compounds. By examining, various compounds exhibit different degree-based TIs, analysts can pinpoint the treatments that are most efficient for specific types of cancer. This paper specifically delves into the topological indices (TIs) implementations in forecasting the biological and physical attributes of innovative compounds utilized in addressing cancer through therapeutic interventions. The analysis being conducted to derivatives of sulfonamides, namely, 4-[(2,4-dichlorophenylsulfonamido)methyl]cyclohexanecarboxylic acid (1), ethyl 4-[(naphthalene-2-sulfonamido)methyl]cyclohexanecarboxylate (2), ethyl 4-[(2,5-dichlorophenylsulfonamido)methyl]cyclohexanecarboxylate (3), 4-[(naphthalene-2-sulfonamido)methyl]cyclohexane-1-carboxylic acid (4) and (2S)-3-methyl-2-(naphthalene-1-sulfonamido)-butanoic acid (5), is performed by utilizing edge partitioning for the computation of degree-based graph descriptors. Subsequently, a linear regression-based model is established to forecast characteristics, like, melting point and formula weight in a quantitative structure-property relationship. The outcomes emphasize the effectiveness or capability of topological indices as a valuable asset for inventing and creating of compounds within the realm of cancer therapy.

## 1 Introduction

Sulfa drugs, commonly known as sulfonamides, encompass a significant functional group exhibiting distinctive biological activities (Boufas et al., 2014). Apart from medicinal applications as antibacterial agent, they are also widely applied in agriculture. Various derivatives of sulfonamides are applied as enzyme inhibitors (Zhong et al., 2004) such as carbonic anhydrase (Winum et al., 2006), metalloproteinase (Cheng et al., 2008), and serine protease (Groutas et al., 2001; Supuran and Scozzafava, 2002). Celecoxib (Penning et al., 1997) and valdecoxib (Talley et al., 2000) are two sulfonamide moieties that act as cyclooxygenase inhibitors. In addition, some sulfonamide compounds show different therapeutic applications in diuretics (Burger and Abraham, 2003), hypoglycemia, HIV protease (Markgren et al., 2002) as phosphodiesterase-5 inhibitor (Rotella, 2002) and in cancer chemotherapy (Crespo et al., 2010). Recently, esters derived from sulfonamides have received considerable attention due to their potential use as cell proliferation inhibitors (Das et al., 2004). Due to the versatile applications of sulfonamides and their derivatives (Danish et al., 2019) in medicinal field, we report on five compounds, 4-[(2, 4-dichloro- phenylsulfonamido)methyl]cyclohexanecarboxylic acid (1), ethyl 4-((naphthalene-2-sulfonamido)-methyl)cyclohexanecarboxylate (2), ethyl 4-[(2, 5-dichlorophenylsulfonamido)methyl]cyclo-hexanecarboxylate (3) (Danish et al., 2021), 4-[(naphthalene-2-sulfonamido)methyl]cyclohexane-1-carboxylic acid (4) (Danish et al., 2015a), and (2S)-3-methyl-2-(naphthalene-1-sulfonamido)-butanoic acid (5) (Danish et al., 2015b).

The calculation of topological indices (TIs) for the mentioned compounds includes using their chemical configurations as well as depicting their molecular compositions. The notion of topological indices (TIs), pioneered by H. Wiener in 1947 (Wiener, 1947), proves to be a valuable tool for characterizing the constructions of molecular graphs (Ramírez Alfaro, 2022). These indices provide quantitative measures that help describe the connectivity patterns within the compounds, offering insights into the structural features of the molecules. This information is crucial for understanding the relationships between molecular structures and properties, particularly in the context of anti-cancer compounds. Some of the topological indices we have discussed include first and second Zagreb (M_1_, M_2_ and ^m^M_2_), harmonic (H), hyper Zagreb (HM), forgotten (F), reciporcal Randic (RR), Randic (RA), sum connectivity (S), geometric arithmetic (GA) and atom bond connectivity (ABC) index.

Numerical values, obtained from the molecular formulas of the compounds (Mohammed et al., 2016), are provided by these topological indices. These indices utilize mathematical algorithms based on the structural information encoded in the molecular graphs, offering quantitative insights into the compounds’ topological features and connectivity patterns. Some interesting results on topological indices are characterized in (Natarajan et al., 2022; Zaman and He, 2022; Ullah et al., 2023a; Ullah et al., 2023b; Yan et al., 2023; Zaman et al., 2023; Arockiaraj et al., 2024a; Hayat et al., 2024a; Arockiaraj et al., 2024b; Hayat et al., 2024b; Arockiaraj et al., 2024c; Chidambaram et al., 2024). This numerical representation facilitates the characterization and comparison of different molecular structures, contributing to the understanding of their properties and potential activities, including anti-cancer effects.

Molecular descriptors find applications in diverse fields, including biology and mathematics (Aslam et al., 2017; Gutman et al., 2018). In this study, linear regression (Hosamani et al., 2017) employed to calculate various properties of these compounds, such as melting point (MP) and formula weight (FW), aiming to establish correlations between topological indices (TIs) and physicochemical characterizations. Linear regression analysis may be extended to higher-order regression models by using higher-order predictor variables. In basic linear regression, a linear equation represents the connection between predictors and response variables. To represent nonlinear connections, higher-order regression models include words like squared, cubed, and interaction. Leveraging the derived correlations, QSPR modeling (Duchowicz et al., 2008) will be conducted to accurately estimate the physical and chemical characteristics of the compounds (Hansen and Jurs, 1988). The significance of employing degree-based indices for QSPR analysis stems from their simplicity, resilience, interpretability, computational efficiency, adaptability, and compatibility with graph-based approaches. These indices give useful insights into the structural aspects of molecules and can help to construct predictive models for a variety of chemical attributes.

Numerous uses exist for topological indices (TIs) in the development of compounds. By examining the molecular structures graphs utilize these indices, analysts can point-out the most efficacious compounds (Shanmukha et al., 2022) for particular types of cancer and anticipate the toxicity and potential side effects of the compounds and contribute to the invention and creation of novel compounds. In summary, the using of topological indices (TIs) in compound exploitation has the capability to significantly advance the treatment of cancer and deepen our comprehension of molecular structures.

Compounds’ molecular structures are depicted as graphs, with atoms as vertices and the connecting bonds as edges. The graph $G\left(V,E\right)$ is a straightforward, limited, and related depiction of the composition of the compound, with V represents the collections of vertices and E as edges, respectively. In graphical theory, the degree of node in a graph, represented by “du,” signifies the count of nodes adjacent to it. In chemistry, the valence of a compound corresponds to the degree of its associated node in the graph. The presented table (Table 1) includes information about topological indices, their notations, formulas, and the years in which they were introduced.

**TABLE 1:** The considered topological indices, notations and formulas.

Topological indices	Notation	Formula	Introduced
**First Zagreb Index**	M_1_(G)	$\sum_{uv\in E\left(G\right)}\left(du+dv\right),\sum_{uv\in E\left(G\right)}\left(du\times dv\right)$	Gutman and Trinajstic’ (Gutman and Trinajstić, 1972)
	M_2_(G)		
**Second Zagreb Index**	^m^M_2_(G)	$\sum_{uv\in E\left(G\right)}\left(\frac{1}{du+dv}\right)$	Gutman, I. and Polansky, O. E. (Gutman and Polansky, 2012)
**Harmonic index**	H(G)	$\sum_{uv\in E\left(G\right)}\left(\frac{2}{du+dv}\right)$	Graffiti (Fajtlowicz, 1988)
**Hyper Zagreb index**	HM(G)	$\sum_{uv\in E\left(G\right)}{\left(du+dv\right)}^{2}$	G.H. Shirdel, H. Rezapour and A.M. Sayadi (Shirdel et al., 2013)
**Forgotten index**	F(G)	$\left.\sum_{uv\in E\left(G\right)}\left[du^{2}\right.+dv^{2}\right]$	Furtulaand Gutman, 2015 (Furtula and Gutman, 2015)
**Reciprocal Randic Index**	RR(G)	$\sum_{uv\in E\left(G\right)}\sqrt{du\times dv}$	In 2014, Gutman, I., Furtula, B., and Elphick, C. (Gutman et al., 2014)
**Randic Index**	RA(G)	$\sum_{uv\in E\left(G\right)}\sqrt{\frac{1}{du\times dv}}$	In 1975, Million Randic (Farahani, 2013)
**Sum Connectivity**	S(G)	$\sum_{uv\in E\left(G\right)}\sqrt{\frac{1}{du+dv}}$	Zhou and Trinajstić (Farahani, 2013)
**Geometric Arithmetic index**	GA(G)	$\sum_{uv\in E\left(G\right)}2\frac{\sqrt{du\times dv}}{du+dv}$	Shegehall and Kanabur (Vukičević and Furtula, 2009)
**Atom Bond Connectivity Index**	ABC(G)	$\sum_{uv\in E\left(G\right)}\sqrt{\frac{du+dv-2}{du\times dv}}$	Ernesto Estrade, 1998 (Das et al., 2011)

## 2 Results and discussions

Molecular descriptors find extensive applications in medicine, particularly in the domains of inventing and creating compounds. In the context of cancer treatment, the application of topological indices (TIs) becomes crucial for identifying potential compound candidates possessing the targeted physicochemical properties. Applying topology-related degree indices to compounds for the treatment of cancer allows for a deeper understanding of their structural characteristics and facilitates the correlation of these features with their biological activity. This approach provides valuable insights for the targeted design and discovery of compounds tailored for effective blood cancer treatment.

The quantitative structure-property relationship (QSPR) modeling approach proves beneficial for analyzing the relationship among molecular attributes and the physico-chemical characteristics of compounds for addressing cancer. These aspects are instrumental in estimating the physical and chemical characteristics of newly discovered compounds, candidates according to their structural attributes, thus enhancing the efficiency of compound discovered. By leveraging QSPR modeling, researchers can gain valuable insights into how specific structural elements influence the properties of compounds, facilitating a more informed and targeted approach to identifying promising candidates for cancer treatment.

**FIGURE 1:**
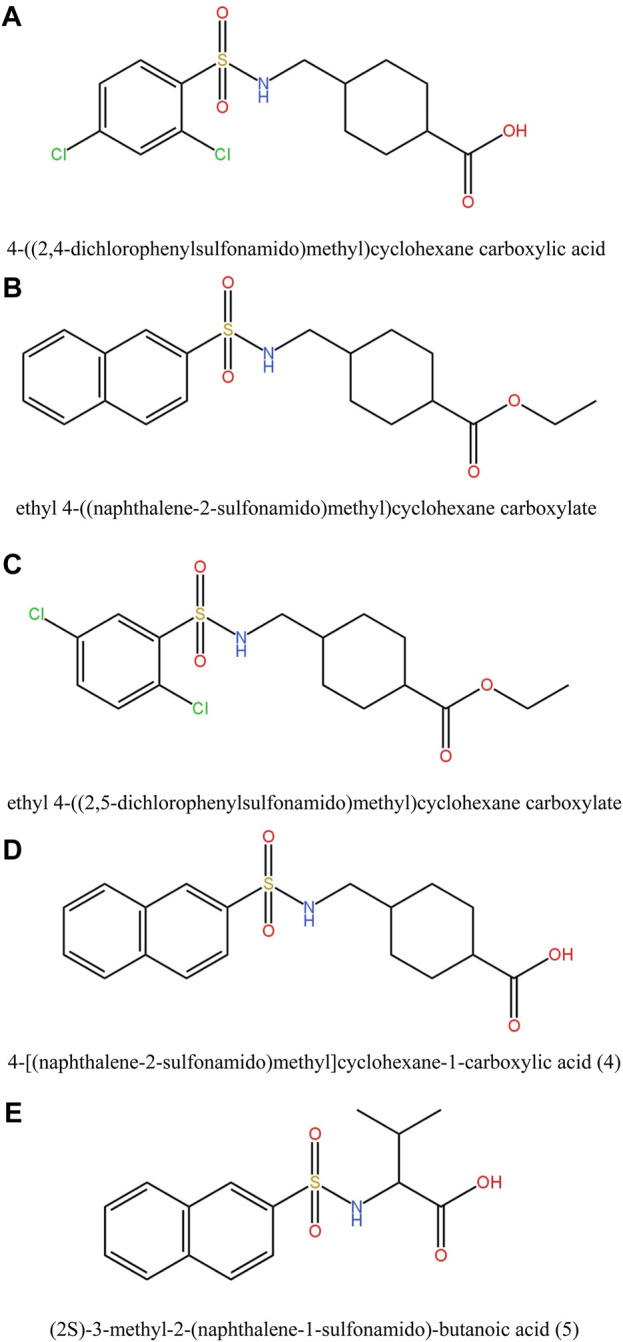
Molecular Structures of derivatives of Sulfonamides as given in **(A)–(E)**.

**FIGURE 2:**
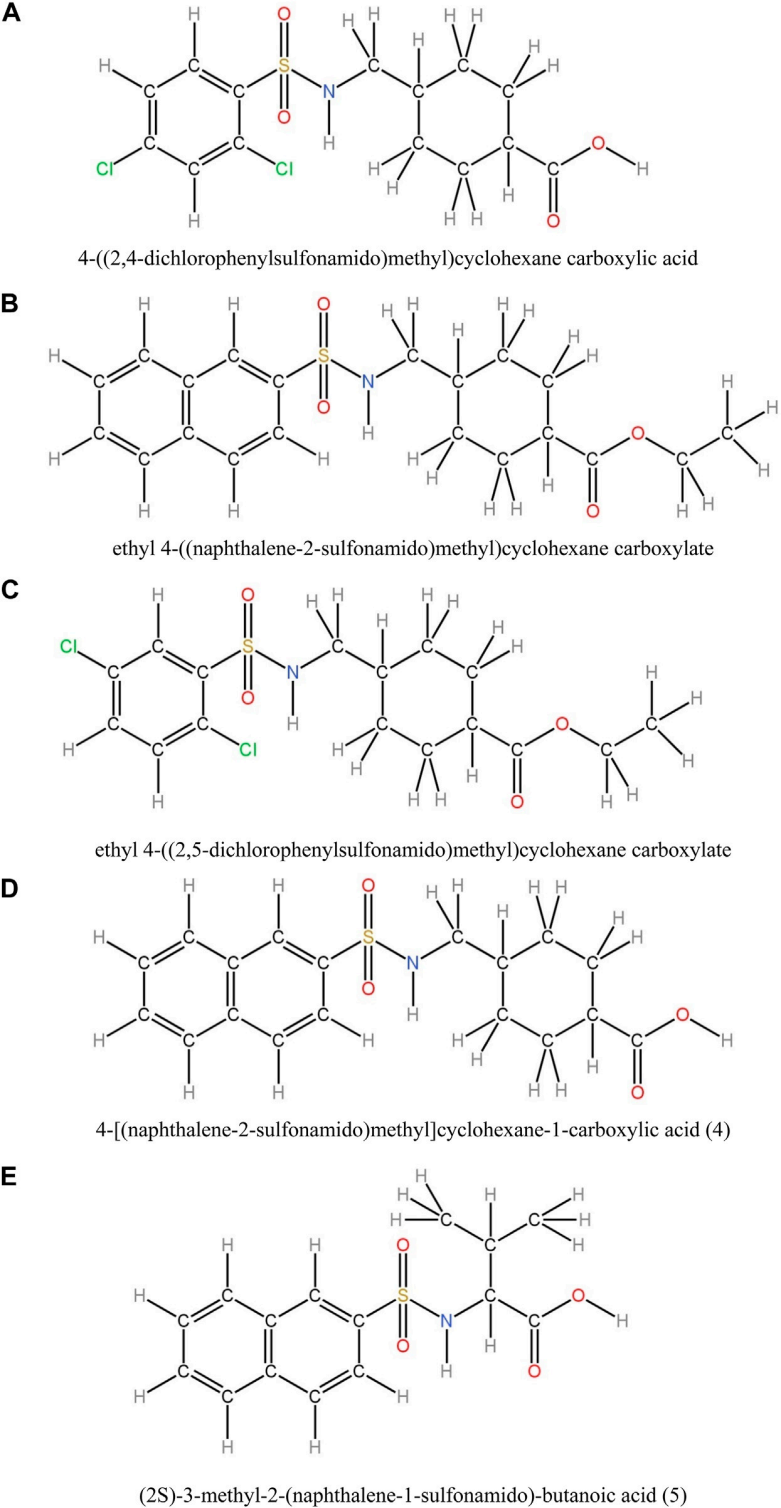
Chemical Structures of derivatives of Sulfonamides as given in **(A)–(E)**.

In this study, various compounds employed in cancer treatment underwent analysis utilizing topological indices and QSPR modeling. The compounds examined encompassed 4-[(2,4-dichlorophenylsulfonamido)methyl]cyclohexanecarboxylic acid, ethyl 4-((naphthalene-2-sulfona-mido)methyl)cyclohexanecarboxylate, ethyl 4-[(2,5-dichlorophenylsulfonamido)methyl]cyclo-hexanecarboxylate, 4-[(naphthalene-2-sulfonamido)methyl]cyclohexane-1-carboxylic acid and (2S)-3-methyl-2-(naphthalene-1-sulfonamido)-butanoic acid, as illustrated by their molecular structure in Figure 1 and chemical structure in Figure 2. Employing degree-based topological indices on these compounds enabled the calculation of numerical values, facilitating the correlation of these indices with their respective physicochemical properties.

The findings from the current analysis hold significant implications for advancing the creation of novel compounds in the treatment of cancer. Identifying the structural attributes and physicochemical properties of effective compounds provides valuable insights for designing new compounds with comparable attributes and potentially enhanced efficacy.

Moreover, this approach supports the enhancement of existing compounds through strategic modifications to their structural features, aimed at improving physical and chemical properties and augmenting its efficacy in the treatment of cancer. Analytical regression played a crucial role in the calculations conducted in this study.

### 2.1 Model of regression

The model of regression serves as a valuable tool in establishing relation between molecular attributes and the physico-chemical properties of compounds employed for addressing cancer. The results indicate a robust relationship between topological indices (TIs), the physical and chemical properties of these compounds.

The utilization of topological indices (TIs) in the context of cancer research is multifaceted. These descriptors prove instrumental in analyzing the structures of various compounds used in cancer treatment, spanning chemotherapeutic agents, targeted therapies, hormonal therapies, and immunotherapies. Analyzing topological indices in compound design aids in identifying new compounds and optimizing those already in existence. For instance, molecular descriptors enable the prediction of the efficacy of novel compounds in the treatment of cancer by scrutinizing their inherent structure.

Furthermore, Topological indices play a crucial role in determining the mechanism of action of compounds for addressing cancer, offering insightful understandings into the biological processes, underlying these compounds. In summary, the integration of topological indices (TIs) in cancer studies holds the potential to unearth new compounds and enhance the effectiveness of those already in use. The acquired outcomes undergo rigorous testing through mathematical expression 1.

$$P=A+B\left(TI\right)$$ (1)

Here, the sign P signifies a parameter associated with the physical and chemical properties of a compound. TI stands for some topological indices, whereas A, B represent the coefficients of regression utilized in this observation. With the aid of a linear QSPR model, the eleven TIs of potential cancer treatments are examined, as well as their physical characteristics. Using (1), we create a linear regression model for TIs of the potential compounds listed below.

Theorem 1 Let G_1_ denotes the 4-[(2, 4-dichlorophenylsulfonamido)methyl]cyclohexane carboxylic acid, then the following axioms holds;

i. M_1_ (*G*_1_) = 136
ii. M_2_ (*G*_1_) = 162
iii. ^m^M_2_ (*G*_1_) = 5.58
iv. H (*G*_1_) = 11.18
v. HM(*G*_1_) = 714
vi. F (*G*_1_) = 390
vii. RR (*G*_1_) = 64.17
viii. RA (*G*_1_) = 12.01
ix. S (*G*_1_) = 12.22
x. GA (*G*_1_) = 25.38
xi. ABC(*G*_1_) = 19.82

**Proof.** Let G_1_ belongs to 4-[(2,4-dichlorophenylsulfonamido)methyl]cyclohexane carboxylic acid, with the edge set represented as E and E^1^ (u, v) denoting the set of edges in G_1_ that adds degrees vertices “u” and “v,” the frequencies are provided as follows:

|*E* ^1^_1,3_ | = 4, |*E* ^1^_2,2_ | = 3, |*E* ^1^_2,3_ | = 9, |*E* ^1^_3,4_ | = 4, |*E* ^1^_1,2_ | = 1, |*E* ^1^_3,3_ | = 2, |*E* ^1^_1,4_ | = 4. Then.

i) By applying the first Zagreb index (M_1_) and the provided edge partitions E^1^ (u, v), we obtain:

$$\begin{array}{l} M_{1}\left(G_{1}\right)=4\left(1+3\right)+3\left(2+2\right)+9\left(2+3\right)+4\left(3+4\right)+1\left(1+2\right)+2\left(3+3\right)+4\left(1+4\right)=136 \end{array}$$

ii) By applying new version first Zagreb index (M_2_) and the provided edge partitions E^1^ (u, v), we obtain:

$$\begin{array}{l} M_{2}\left(G_{1}\right)=4\left(1\times 3\right)+3\left(2\times 2\right)+9\left(2\times 3\right)+4\left(3\times 4\right)+1\left(1\times 2\right)+2\left(3\times 3\right)+4\left(1\times 4\right)=162 \end{array}$$

iii) By applying second Zagreb index (^m^M_2_) and the provided edge partitions E^1^ (u, v), we obtain:

$$\begin{array}{l} M_{2}^{m}\left(G_{1}\right)=4\left(\frac{1}{1+3}\right)+3\left(\frac{1}{2+2}\right)+9\left(\frac{1}{2+3}\right)+4\left(\frac{1}{3+4}\right)+1\left(\frac{1}{1+2}\right)+2\left(\frac{1}{3+3}\right)+4\left(\frac{1}{1+4}\right)=5.58 \end{array}$$

iv) By applying harmonic index (H) and the provided edge partitions E^1^ (u, v), we obtain:

$$\begin{array}{l} H\left(G_{1}\right)=4\left(\frac{2}{1+3}\right)+3\left(\frac{2}{2+2}\right)+9\left(\frac{2}{2+3}\right)+4\left(\frac{2}{3+4}\right)+1\left(\frac{2}{1+2}\right)+2\left(\frac{2}{3+3}\right)+4\left(\frac{2}{1+4}\right)=11.18 \end{array}$$

v) By applying hyper Zagreb index (HM) and the provided edge partitions E^1^ (u, v), we obtain:

$$\begin{array}{l} HM\left(G_{1}\right)=4{\left(1+3\right)}^{2}+3{\left(2+2\right)}^{2}+9{\left(2+3\right)}^{2}+4{\left(3+4\right)}^{2}+1{\left(1+2\right)}^{2}+2{\left(3+3\right)}^{2}+4{\left(1+4\right)}^{2}=714 \end{array}$$

vi) By applying forgotten index (F) and the provided edge partitions E^1^ (u, v), we obtain:

$$\begin{array}{l} F\left(G_{1}\right)=4\left(1^{2}+3^{2}\right)+3\left(2^{2}+2^{2}\right)+9\left(2^{2}+3^{2}\right)+4\left(3^{2}+4^{2}\right)+1\left(1^{2}+2^{2}\right)+2\left(3^{2}+3^{2}\right)+4\left(1^{2}+4^{2}\right)=390 \end{array}$$

vii) By applying reciprocal randic index (RR) and the provided edge partitions E^1^ (u, v), we obtain:

$$\begin{array}{l} RR\left(G_{1}\right)=4\sqrt{1\times 3}+3\sqrt{2\times 2}+9\sqrt{2\times 3}+4\sqrt{3\times 4}+1\sqrt{1\times 2}+2\sqrt{3\times 3}+4\sqrt{1\times 4}=64.17 \end{array}$$

viii) By applying randic index (RA) and the provided edge partitions E^1^ (u, v), we obtain:

$$\begin{array}{l} RA\left(G_{1}\right)=4\sqrt{\frac{1}{1\times 3}}+3\sqrt{\frac{1}{2\times 2}}+9\sqrt{\frac{1}{2\times 3}}+4\sqrt{\frac{1}{3\times 4}}+1\sqrt{\frac{1}{1\times 2}}+2\sqrt{\frac{1}{3\times 3}}+4\sqrt{\frac{1}{1\times 4}}=12.01 \end{array}$$

ix) By applying sum connectivity index (S) and the provided edge partitions E^1^ (u, v), we obtain:

$$\begin{array}{l} S\left(G_{1}\right)=4\sqrt{\frac{1}{1+3}}+3\sqrt{\frac{1}{2+2}}+9\sqrt{\frac{1}{2+3}}+4\sqrt{\frac{1}{3+4}}+1\sqrt{\frac{1}{1+2}}+2\sqrt{\frac{1}{3+3}}+4\sqrt{\frac{1}{1+4}}=12.22 \end{array}$$

x) By applying geometric arithmetic index (GA) and the provided edge partitions E^1^ (u, v), we obtain:

$$\begin{array}{l} GA\left(G_{1}\right)=2\left(4\frac{\sqrt{1\times 3}}{1+3}\right)+2\left(3\frac{\sqrt{2\times 2}}{2+2}\right)+2\left(9\frac{\sqrt{2\times 3}}{2+3}\right)+2\left(4\frac{\sqrt{3\times 4}}{3+4}\right)+2\left(1\frac{\sqrt{1\times 2}}{1+2}\right)+2\left(2\frac{\sqrt{3\times 3}}{3+3}\right)+2\left(4\frac{\sqrt{1\times 4}}{1+4}\right)=25.38 \end{array}$$

xi) By applying atom bond connectivity index (ABC) and the provided edge partitions E^1^ (u, v), we obtain:

$$\begin{array}{l} ABC\left(G_{1}\right)=4\sqrt{\frac{1+3-2}{1\times 3}}+3\sqrt{\frac{2+2-2}{2\times 2}}+9\sqrt{\frac{2+3-2}{2\times 3}}+4\sqrt{\frac{3+4-2}{3\times 4}}+1\sqrt{\frac{1+2-2}{1\times 2}}+2\sqrt{\frac{3+3-2}{3\times 3}}\\ +4\sqrt{\frac{1+4-2}{1\times 4}}=19.82 \end{array}$$

Theorem 2 Let G_2_ denotes the ethyl 4-[(naphthalene-2-sulfonamido)methyl]cyclohexane carboxylate, then the following axioms satisfied for G_2._

i. M_1_ (*G*_2_) = 186
ii. M_2_ (*G*_2_) = 222
iii. ^m^M_2_ (*G*_2_) = 7.19
iv. H (*G*_2_) = 14.39
v. HM(*G*_2_) = 996
vi. F (*G*_2_) = 552
vii. RR (*G*_2_) = 87.09
viii. RA (*G*_2_) = 15.56
ix. S (*G*_2_) = 16.03
x. GA (*G*_2_) = 33.61
xi. ABC(*G*_2_) = 26.67

**Proof.** Let G_2_ belongs to ethyl 4-[(naphthalene-2-sulfonamido)methyl]cyclohexane carboxylate with the edge set represented as E^2^ and E^2^ (u, v) denoting the set of edges in G_2_ that adds degrees vertices “u” and “v,” the frequencies are provided as follows:

|*E* ^2^_(2,2)_| = 6, |*E* ^2^_(2,3)_| = 11, |*E* ^2^_(3,4)_| = 4, |*E* ^2^_(1,4)_| = 9, |*E* ^2^_(1,3)_| = 2, |*E* ^2^_(3,3)_| = 2, |*E* ^2^_(2,4)_ | = 1, |*E* ^2^_(4,4)_| = 1. Then.

i) Applying first Zagreb index (M_1_) and the provided edge partition E^2^ (u, v), we obtain:

$$\begin{array}{l} M_{1}\left(G_{2}\right)=6\left(2+2\right)+11\left(2+3\right)+4\left(3+4\right)+9\left(1+4\right)+2\left(1+3\right)+2\left(3+3\right)+1\left(2+4\right)+1\left(4+4\right)=186 \end{array}$$

ii) By applying new version first Zagreb index (M_2_) and the provided edge partition E^2^ (u, v), we obtain:

$$\begin{array}{l} M_{2}\left(G_{2}\right)=6\left(2\times 2\right)+11\left(2\times 3\right)+4\left(3\times 4\right)+9\left(1\times 4\right)+2\left(1\times 3\right)+2\left(3\times 3\right)+1\left(2\times 4\right)+1\left(4\times 4\right)=222 \end{array}$$

iii) By applying second Zagreb index (^m^M_2_) and the provided edge partition E^2^ (u, v), we obtain:

M2mG2=612+2+1112+3+413+4+911+4+211+3+213+3+112+4+114+4=7.19

iv) By applying harmonic index (H) and the provided edge partition E^2^ (u, v), we obtain:

$$\begin{array}{l} H\left(G_{2}\right)=6\left(\frac{2}{2+2}\right)+11\left(\frac{2}{2+3}\right)+4\left(\frac{2}{3+4}\right)+9\left(\frac{2}{1+4}\right)+2\left(\frac{2}{1+3}\right)+2\left(\frac{2}{3+3}\right)+1\left(\frac{2}{2+4}\right)+1\left(\frac{2}{4+4}\right)=14.39 \end{array}$$

v) By applying hyper Zagreb index (HM) and the provided edge partition E^2^ (u, v), we obtain:

$$\begin{array}{l} HM\left(G_{2}\right)=6{\left(2+2\right)}^{2}+11{\left(2+3\right)}^{2}+4{\left(3+4\right)}^{2}+9{\left(1+4\right)}^{2}+2{\left(1+3\right)}^{2}+2{\left(3+3\right)}^{2}+1{\left(2+4\right)}^{2}+1{\left(4+4\right)}^{2}=996 \end{array}$$

vi) By applying forgotten index (F) and the provided edge partition E^2^ (u, v), we obtain:

$$\begin{array}{l} F\left(G_{2}\right)=6\left(2^{2}+2^{2}\right)+11\left(2^{2}+3^{2}\right)+4\left(3^{2}+4^{2}\right)+9\left(1^{2}+4^{2}\right)+2\left(1^{2}+3^{2}\right)+2\left(3^{2}+3^{2}\right)+1\left(2^{2}+4^{2}\right)+1\left(4^{2}+4^{2}\right)=552 \end{array}$$

vii) By applying reciprocal randic index (RR) and the provided edge partition E^2^ (u, v), we obtain:

$$\begin{array}{l} RR\left(G_{2}\right)=6\sqrt{2\times 2}+11\sqrt{2\times 3}+4\sqrt{3\times 4}+9\sqrt{1\times 4}+2\sqrt{1\times 3}+2\sqrt{3\times 3}+1\sqrt{2\times 4}+1\sqrt{4\times 4}=87.09 \end{array}$$

viii) By applying randic index (RA) and the provided edge partition E^2^ (u, v), we obtain:

$$\begin{array}{l} RA\left(G_{2}\right)=6\sqrt{\frac{1}{2\times 2}}+11\sqrt{\frac{1}{2\times 3}}+4\sqrt{\frac{1}{3\times 4}}+9\sqrt{\frac{1}{1\times 4}}+2\sqrt{\frac{1}{1\times 3}}+2\sqrt{\frac{1}{3\times 3}}+1\sqrt{\frac{1}{2\times 4}}+1\sqrt{\frac{1}{4\times 4}}=15.56 \end{array}$$

ix) By applying sum connectivity index (S) and the provided edge partition E^2^ (u, v), we obtain:

$$\begin{array}{l} S\left(G_{2}\right)=6\sqrt{\frac{1}{2+2}}+11\sqrt{\frac{1}{2+3}}+4\sqrt{\frac{1}{3+4}}+9\sqrt{\frac{1}{1+4}}+2\sqrt{\frac{1}{1+3}}+2\sqrt{\frac{1}{3+3}}+1\sqrt{\frac{1}{2+4}}+1\sqrt{\frac{1}{4+4}}=16.03 \end{array}$$

x) By applying geometric arithmetic index (GA) and the provided edge partition E^2^ (u, v), we obtain:

$$\begin{array}{l} GA\left(G_{2}\right)=2\left(6\frac{\sqrt{2\times 2}}{2+2}\right)+2\left(11\frac{\sqrt{2\times 3}}{2+3}\right)+2\left(4\frac{\sqrt{3\times 4}}{3+4}\right)+2\left(9\frac{\sqrt{1\times 4}}{1+4}\right)+2\left(2\frac{\sqrt{1\times 3}}{1+3}\right)+2\left(2\frac{\sqrt{3\times 3}}{3+3}\right)\\ +2\left(1\frac{\sqrt{2\times 4}}{2+4}\right)+2\left(1\frac{\sqrt{4\times 4}}{4+4}\right)=33.61 \end{array}$$

xi) By applying atom bond connectivity index (ABC) and the provided edge partition E^2^ (u, v), we obtain:

$$\begin{array}{l} ABC\left(G_{2}\right)=6\sqrt{\frac{2+2-2}{2\times 2}}+11\sqrt{\frac{2+3-2}{2\times 3}}+4\sqrt{\frac{3+4-2}{3\times 4}}+9\sqrt{\frac{1+4-2}{1\times 4}}+2\sqrt{\frac{1+3-2}{1\times 3}}+2\sqrt{\frac{3+3-2}{3\times 3}}\\ +1\sqrt{\frac{2+4-2}{2\times 4}}+1\sqrt{\frac{4+4-2}{4\times 4}}=6.67 \end{array}$$

Theorem 3 Let G_3_ denotes the ethyl 4-[(2, 5-dichlorophenylsulfonamido)methyl]cyclohexane carboxylate, then the following axioms satisfied for G_3._

i. M_1_ (*G*_3_) = 172
ii. M_2_ (*G*_3_) = 204
iii. ^m^M_2_ (*G*_3_) = 6.54
iv. H (*G*_3_) = 13.09
v. HM(*G*_3_) = 930
vi. F (*G*_3_) = 522
vii. RR (*G*_3_) = 79.67
viii. RA (*G*_3_) = 14.4
ix. S (*G*_3_) = 14.63
x. GA (*G*_3_) = 30.38
xi. ABC(*G*_3_) = 24.76

**Proof.** Let G_3_ belongs to ethyl 4-[(2,5-dichlorophenylsulfonamido)methyl]cyclohexane carboxylate with the edge set represented as E^3^ and E^3^ (u,v) denoting the set of edges in G_3_ that adds degrees vertices “u” and “v,” the frequencies are provided as follows:

|*E* ^3^_(1,3)_| = 4, |*E* ^3^_(2,3)_| = 9, |*E* ^3^_(2,2)_| = 3, |*E* ^3^_(1,4)_| = 9, |*E* ^3^_(3,4)_| = 4, |*E* ^3^_(4,4)_| = 1, |*E* ^3^_(3,3)_ | = 2, |*E* ^3^_(2,4)_| = 1. Then.

i) By applying first Zagreb index (M_1_) and the provided edge partitions E^3^ (u, v), we obtain:

$$\begin{array}{l} M_{1}^{m}\left(G_{3}\right)=4\left(1+3\right)+9\left(2+3\right)+3\left(2+2\right)+9\left(1+4\right)+4\left(3+4\right)+1\left(4+4\right)+2\left(3+3\right)+1\left(2+4\right)=172 \end{array}$$

ii) By applying new version first Zagreb index (M_2_) and the provided edge partitions E^3^ (u, v), we obtain:

$$\begin{array}{l} M_{2}\left(G_{3}\right)=4\left(1\times 3\right)+9\left(2\times 3\right)+3\left(2\times 2\right)+9\left(1\times 4\right)+4\left(3\times 4\right)+1\left(4\times 4\right)+2\left(3+3\right)+1\left(2\times 4\right)=204 \end{array}$$

iii) By applying second Zagreb index (^m^M_2_) and the provided edge partitions E^3^ (u, v), we obtain:

$$\begin{array}{l} M_{2}\left(G_{3}\right)=4\left(\frac{1}{1+3}\right)+9\left(\frac{1}{2+3}\right)+3\left(\frac{1}{2+2}\right)+9\left(\frac{1}{1+4}\right)+4\left(\frac{1}{3+4}\right)+1\left(\frac{1}{4+4}\right)+2\left(\frac{1}{3+3}\right)+1\left(\frac{1}{2+4}\right)=6.54 \end{array}$$

iv) By applying harmonic index (H) and the provided edge partitions E^3^ (u, v), we obtain:

$$\begin{array}{l} H\left(G_{3}\right)=4\left(\frac{2}{1+3}\right)+9\left(\frac{2}{2+3}\right)+3\left(\frac{2}{2+2}\right)+9\left(\frac{2}{1+4}\right)+4\left(\frac{2}{3+4}\right)+1\left(\frac{2}{4+4}\right)+2\left(\frac{2}{3+3}\right)+1\left(\frac{2}{2+4}\right)=13.09 \end{array}$$

v) By applying hyper Zagreb index (HM) and the provided edge partitions E^3^ (u, v), we obtain:

$$\begin{array}{l} HM\left(G_{3}\right)=4{\left(1+3\right)}^{2}+9{\left(2+3\right)}^{2}+3{\left(2+2\right)}^{2}+9{\left(1+4\right)}^{2}+4{\left(3+4\right)}^{2}+1{\left(4+4\right)}^{2}+2{\left(3+3\right)}^{2}+1{\left(2+4\right)}^{2}=930 \end{array}$$

vi) By applying forgotten index (F) and the provided edge partitions E^3^ (u, v), we obtain:

$$\begin{array}{l} F\left(G_{3}\right)=4\left(1^{2}+3^{2}\right)+9\left(2^{2}+3^{2}\right)+3\left(2^{2}+2^{2}\right)+9\left(1^{2}+4^{2}\right)+4\left(3^{2}+4^{2}\right)+1\left(4^{2}+4^{2}\right)+2\left(3^{2}+3^{2}\right)+1\left(2^{2}+4^{2}\right)=522 \end{array}$$

vii) By applying reciprocal randic index (RR) and the provided edge partitions E^3^ (u, v), we obtain:

$$\begin{array}{l} RR\left(G_{3}\right)=4\sqrt{1\times 3}+9\sqrt{2\times 3}+3\sqrt{2\times 2}+9\sqrt{1\times 4}+4\sqrt{3\times 4}+1\sqrt{4\times 4}+2\sqrt{3\times 3}+1\sqrt{2\times 4}=79.67 \end{array}$$

viii) By applying randic index (RA) and the provided edge partitions E^3^ (u, v), we obtain:

$$\begin{array}{l} RA\left(G_{3}\right)=4\sqrt{\frac{1}{1\times 3}}+9\sqrt{\frac{1}{2\times 3}}+3\sqrt{\frac{1}{2\times 2}}+9\sqrt{\frac{1}{1\times 4}}+4\sqrt{\frac{1}{3\times 4}}+1\sqrt{\frac{1}{4\times 4}}+2\sqrt{\frac{1}{3\times 3}}+1\sqrt{\frac{1}{2\times 4}}=14.4 \end{array}$$

ix) By applying sum connectivity index (S) and the provided edge partitions E^3^ (u, v), we obtain:

$$\begin{array}{l} S\left(G_{3}\right)=4\sqrt{\frac{1}{1+3}}+9\sqrt{\frac{1}{2+3}}+3\sqrt{\frac{1}{2+2}}+9\sqrt{\frac{1}{1+4}}+4\sqrt{\frac{1}{3+4}}+1\sqrt{\frac{1}{4+4}}+2\sqrt{\frac{1}{3+3}}+1\sqrt{\frac{1}{2+4}}=14.63 \end{array}$$

x) By applying geometric arithmetic index (GA) and the provided edge partitions E^3^ (u, v), we obtain:

$$\begin{array}{l} GA\left(G_{3}\right)=2\left(4\frac{\sqrt{1\times 3}}{1+3}\right)+2\left(9\frac{\sqrt{2\times 3}}{2+3}\right)+2\left(3\frac{\sqrt{2\times 2}}{2+2}\right)+2\left(9\frac{\sqrt{1\times 4}}{1+4}\right)+2\left(4\frac{\sqrt{3\times 4}}{3+4}\right)+2\left(1\frac{\sqrt{4\times 4}}{4+4}\right)\\ +2\left(2\frac{\sqrt{3\times 3}}{3+3}\right)+2\left(1\frac{\sqrt{2\times 4}}{2+4}\right)=30.38 \end{array}$$

xi) By applying atom bond connectivity index (ABC) and the provided edge partitions E^3^ (u, v), we obtain:

$$\begin{array}{l} ABC\left(G_{3}\right)=4\sqrt{\frac{1+3-2}{1\times 3}}+9\sqrt{\frac{2+3-2}{2\times 3}}+3\sqrt{\frac{2+2-2}{2\times 2}}+9\sqrt{\frac{1+4-2}{1\times 4}}+4\sqrt{\frac{3+4-2}{3\times 4}}+1\sqrt{\frac{4+4-2}{4\times 4}}\\ +2\sqrt{\frac{3+3-2}{3\times 3}}+1\sqrt{\frac{2+4-2}{2\times 4}}=24.76 \end{array}$$

Theorem 4 Let G_4_ denotes the 4-[(naphthalene-2-sulfonamido)methyl]cyclohexane-1-carboxylic acid, then the following axioms satisfied for G_4._

i. M_1_ (*G*_4_) = 150
ii. M_2_ (*G*_4_) = 180
iii. ^m^M_2_ (*G*_4_) = 6.33
iv. H (*G*_4_) = 12.49
v. HM(*G*_4_) = 780
vi. F (*G*_4_) = 420
vii. RR (*G*_4_) = 72.66
viii. RA (*G*_4_) = 13.2
ix. S (*G*_4_) = 13.67
x. GA (*G*_4_) = 28.62
xi. ABC(*G*_4_) = 21.82

**Proof.** Let G_4_ belongs to 4-[(naphthalene-2-sulfonamido)methyl]cyclohexane-1-carboxylic acid with the edge set represented as E^4^ and E^4^ (u,v) denoting the set of edges in G_4_ that adds degrees vertices “u” and “v,” the frequencies are provided as follows:

|*E* ^4^_1,2_ | = 1, |*E* ^4^_1,3_| = 2, |*E* ^4^_1,4_ | = 4, |*E* ^4^_2,2_ | = 6, |*E* ^4^_2,3_ | = 11, |*E* ^4^_3,3_ | = 2, |*E* ^4^_3,4_ | = 4. Then.

i) By applying first Zagreb index (M_1_) and the provided edge partitions E^4^ (u, v), we obtain:

$$\begin{array}{l} M_{1}\left(G_{4}\right)=2\left(1+3\right)+11\left(2+3\right)+6\left(2+2\right)+4\left(1+4\right)+4\left(3+4\right)+1\left(1+2\right)+2\left(3+3\right)=150 \end{array}$$

ii) By applying new version first Zagreb index (M_2_) and the provided edge partitions E^4^ (u, v), we obtain:

$$\begin{array}{l} M_{2}\left(G_{4}\right)=2\left(1\times 3\right)+11\left(2\times 3\right)+6\left(2\times 2\right)+4\left(1\times 4\right)+4\left(3\times 4\right)+1\left(1\times 2\right)+2\left(3+3\right)=180 \end{array}$$

iii) By applying second Zagreb index (^m^M_2_) and the provided edge partitions E^4^ (u, v), we obtain:

M2mG4=211+3+1112+3+612+2+411+4+413+4+111+2+213+3=6.33

iv) By applying harmonic index (H) and the provided edge partitions E^4^ (u, v), we obtain:

$$\begin{array}{l} H\left(G_{4}\right)=2\left(\frac{2}{1+3}\right)+11\left(\frac{2}{2+3}\right)+6\left(\frac{2}{2+2}\right)+4\left(\frac{2}{1+4}\right)+4\left(\frac{2}{3+4}\right)+1\left(\frac{2}{1+2}\right)+2\left(\frac{2}{3+3}\right)=12.49 \end{array}$$

v) By applying hyper Zagreb index (HM) and the provided edge partitions E^4^ (u, v), we obtain:

$$\begin{array}{l} HM\left(G_{4}\right)=2{\left(1+3\right)}^{2}+11{\left(2+3\right)}^{2}+6{\left(2+2\right)}^{2}+4{\left(1+4\right)}^{2}+4{\left(3+4\right)}^{2}+1{\left(1+2\right)}^{2}+2{\left(3+3\right)}^{2}=780 \end{array}$$

vi) By applying forgotten index (F) and the provided edge partitions E^4^ (u, v), we obtain:

$$\begin{array}{l} F\left(G_{4}\right)=2\left(1^{2}+3^{2}\right)+11\left(2^{2}+3^{2}\right)+6\left(2^{2}+2^{2}\right)+4\left(1^{2}+4^{2}\right)+4\left(3^{2}+4^{2}\right)+1\left(1^{2}+2^{2}\right)+2\left(3^{2}+3^{2}\right)=420 \end{array}$$

vii) By applying reciprocal randic index (RR) and the provided edge partitions E^4^ (u, v), we obtain:

$$\begin{array}{l} RR\left(G_{4}\right)=2\sqrt{1\times 3}+11\sqrt{2\times 3}+6\sqrt{2\times 2}+4\sqrt{1\times 4}+4\sqrt{3\times 4}+1\sqrt{1\times 2}+2\sqrt{3\times 3}=72.66 \end{array}$$

viii) By applying randic index (RA) and the provided edge partitions E^4^ (u, v), we obtain:

$$\begin{array}{l} RA\left(G_{4}\right)=2\sqrt{\frac{1}{1\times 3}}+11\sqrt{\frac{1}{2\times 3}}+6\sqrt{\frac{1}{2\times 2}}+4\sqrt{\frac{1}{1\times 4}}+4\sqrt{\frac{1}{3\times 4}}+1\sqrt{\frac{1}{1\times 2}}+2\sqrt{\frac{1}{3\times 3}}=13.2 \end{array}$$

ix) By applying sum connectivity index (S) and the provided edge partitions E^4^ (u, v), we obtain:

$$\begin{array}{l} S\left(G_{4}\right)=2\sqrt{\frac{1}{1+3}}+11\sqrt{\frac{1}{2+3}}+6\sqrt{\frac{1}{2+2}}+4\sqrt{\frac{1}{1+4}}+4\sqrt{\frac{1}{3+4}}+1\sqrt{\frac{1}{1+2}}+2\sqrt{\frac{1}{3+3}}=13.67 \end{array}$$

x) By applying geometric arithmetic index (GA) and the provided edge partitions E^4^ (u, v), we obtain:

$$\begin{array}{l} GA\left(G_{4}\right)=2\left(2\frac{\sqrt{1\times 3}}{1+3}\right)+2\left(11\frac{\sqrt{2\times 3}}{2+3}\right)+2\left(6\frac{\sqrt{2\times 2}}{2+2}\right)+2\left(4\frac{\sqrt{1\times 4}}{1+4}\right)+2\left(4\frac{\sqrt{3\times 4}}{3+4}\right)+2\left(1\frac{\sqrt{1\times 2}}{1+2}\right)\\ +2\left(2\frac{\sqrt{3\times 3}}{3+3}\right)=28.62 \end{array}$$

xi) By applying atom bond connectivity index (ABC) and the provided edge partitions E^4^ (u, v), we obtain:

$$\begin{array}{l} ABC\left(G_{4}\right)=2\sqrt{\frac{1+3-2}{1\times 3}}+11\sqrt{\frac{2+3-2}{2\times 3}}+6\sqrt{\frac{2+2-2}{2\times 2}}+4\sqrt{\frac{1+4-2}{1\times 4}}+4\sqrt{\frac{3+4-2}{3\times 4}}+1\sqrt{\frac{1+2-2}{1\times 2}}\\ +2\sqrt{\frac{3+3-2}{3\times 3}}=21.82 \end{array}$$

Theorem 5 Let G_5_ denotes the (2S)-3-methyl-2-(naphthalene-1-sulfonamido)-butanoic acid, then the following axioms satisfied for G_5._

i. M_1_ (𝐺_5_) = 170
ii. M_2_ (𝐺_5_) = 211
iii. ^m^M_2_ (𝐺_5_) = 6.35
iv. H (*G*_5_) = 12.71
v. HM(*G*_5_) = 954
vi. F (*G*_5_) = 532
vii. RR (*G*_5_) = 78.86
  viii. RA (*G*_5_) = 13.98
ix. S (*G*_5_) = 14.21
x. GA (*G*_5_) = 29.5
xi. ABC(*G*_5_) = 23.94

**Proof.** Let G_5_ belongs to (2S)-3-methyl-2-(naphthalene-1-sulfonamido)-butanoic acid with the edge set represented as E^5^ and E^5^ (u, v) denoting the set of edges in G_5_ that adds degrees vertices “u” and “v,” the frequencies are provided as follows:

|*E* ^5^_1,2_ | = 1, |*E* ^5^_1,3_| = 2, |*E* ^5^_1,4_ | = 10, |*E* ^5^_2,2_ | = 4, |*E* ^5^_2,3_ | = 7, |*E* ^5^_3,3_ | = 1, |*E* ^5^_3,4_ | = 4, |*E* ^5^_4,4_ | = 3. Then.

i) By applying first Zagreb index (M_1_) and the provided edge partitions E^5^ (u, v), we obtain:

$$\begin{array}{l} M_{1}^{m}\left(G_{5}\right)=2\left(1+3\right)+7\left(2+3\right)+4\left(2+2\right)+10\left(1+4\right)+4\left(3+4\right)+3\left(4+4\right)+1\left(3+3\right)+1\left(1+2\right)=170 \end{array}$$

ii) By applying new version first Zagreb index (M_2_) and the provided edge partitions E^5^ (u, v), we obtain:

$$\begin{array}{l} M_{2}\left(G_{5}\right)=2\left(1\times 3\right)+7\left(2\times 3\right)+4\left(2\times 2\right)+10\left(1\times 4\right)+4\left(3\times 4\right)+3\left(4\times 4\right)+1\left(3+3\right)+1\left(1\times 2\right)=211 \end{array}$$

iii) By applying second Zagreb index (^m^M_2_) and the provided edge partitions E^5^ (u, v), we obtain:

$$\begin{array}{l} M_{2}\left(G_{5}\right)=2\left(\frac{1}{1+3}\right)+7\left(\frac{1}{2+3}\right)+4\left(\frac{1}{2+2}\right)+10\left(\frac{1}{1+4}\right)+4\left(\frac{1}{3+4}\right)+3\left(\frac{1}{4+4}\right)+1\left(\frac{1}{3+3}\right)+1\left(\frac{1}{1+2}\right)=6.35 \end{array}$$

iv) By applying harmonic index (H) and the provided edge partitions E^5^ (u, v), we obtain:

$$\begin{array}{l} H\left(G_{5}\right)=2\left(\frac{2}{1+3}\right)+7\left(\frac{2}{2+3}\right)+4\left(\frac{2}{2+2}\right)+10\left(\frac{2}{1+4}\right)+4\left(\frac{2}{3+4}\right)+3\left(\frac{2}{4+4}\right)+1\left(\frac{2}{3+3}\right)+1\left(\frac{2}{1+2}\right)=12.71 \end{array}$$

v) By applying hyper Zagreb index (HM) and the provided edge partitions E^5^ (u, v), we obtain:

$$\begin{array}{l} HM\left(G_{5}\right)=2{\left(1+3\right)}^{2}+7{\left(2+3\right)}^{2}+4{\left(2+2\right)}^{2}+10{\left(1+4\right)}^{2}+4{\left(3+4\right)}^{2}+3{\left(4+4\right)}^{2}+1{\left(3+3\right)}^{2}+1{\left(1+2\right)}^{2}=954 \end{array}$$

vi) By applying forgotten index (F) and the provided edge partitions E^5^ (u, v), we obtain:

$$\begin{array}{l} F\left(G_{5}\right)=2\left(1^{2}+3^{2}\right)+7\left(2^{2}+3^{2}\right)+4\left(2^{2}+2^{2}\right)+10\left(1^{2}+4^{2}\right)+4\left(3^{2}+4^{2}\right)+3\left(4^{2}+4^{2}\right)+1\left(3^{2}+3^{2}\right)+1\left(1^{2}+2^{2}\right)=532 \end{array}$$

vii) By applying reciprocal randic index (RR) and the provided edge partitions E^5^ (u, v), we obtain:

$$\begin{array}{l} RR\left(G_{5}\right)=2\sqrt{1\times 3}+7\sqrt{2\times 3}+4\sqrt{2\times 2}+10\sqrt{1\times 4}+4\sqrt{3\times 4}+3\sqrt{4\times 4}+1\sqrt{3\times 3}+1\sqrt{1\times 2}=78.86 \end{array}$$

viii) By applying randic index (RA) and the provided edge partitions E^5^ (u, v), we obtain:

$$\begin{array}{l} RA\left(G_{5}\right)=2\sqrt{\frac{1}{1\times 3}}+7\sqrt{\frac{1}{2\times 3}}+4\sqrt{\frac{1}{2\times 2}}+10\sqrt{\frac{1}{1\times 4}}+4\sqrt{\frac{1}{3\times 4}}+3\sqrt{\frac{1}{4\times 4}}+1\sqrt{\frac{1}{3\times 3}}+1\sqrt{\frac{1}{1\times 2}}=13.98 \end{array}$$

ix) By applying sum connectivity index (S) and the provided edge partitions E^5^ (u, v), we obtain:

$$\begin{array}{l} S\left(G_{5}\right)=2\sqrt{\frac{1}{1+3}}+7\sqrt{\frac{1}{2+3}}+4\sqrt{\frac{1}{2+2}}+10\sqrt{\frac{1}{1+4}}+4\sqrt{\frac{1}{3+4}}+3\sqrt{\frac{1}{4+4}}+1\sqrt{\frac{1}{3+3}}+1\sqrt{\frac{1}{1+2}}=14.21 \end{array}$$

x) By applying geometric arithmetic index (GA) and the provided edge partitions E^5^ (u, v), we obtain:

$$\begin{array}{l} GA\left(G_{5}\right)=2\left(2\frac{\sqrt{1\times 3}}{1+3}\right)+2\left(7\frac{\sqrt{2\times 3}}{2+3}\right)+2\left(4\frac{\sqrt{2\times 2}}{2+2}\right)+2\left(10\frac{\sqrt{1\times 4}}{1+4}\right)+2\left(4\frac{\sqrt{3\times 4}}{3+4}\right)+2\left(3\frac{\sqrt{4\times 4}}{4+4}\right)\\ +2\left(1\frac{\sqrt{3\times 3}}{3+3}\right)+2\left(1\frac{\sqrt{1\times 2}}{1+2}\right)=29.5 \end{array}$$

xi) By applying atom bond connectivity index (ABC) and the provided edge partitions E^5^ (u, v), we obtain:

$$\begin{array}{l} ABC\left(G_{5}\right)=2\sqrt{\frac{1+3-2}{1\times 3}}+7\sqrt{\frac{2+3-2}{2\times 3}}+4\sqrt{\frac{2+2-2}{2\times 2}}+10\sqrt{\frac{1+4-2}{1\times 4}}+4\sqrt{\frac{3+4-2}{3\times 4}}+3\sqrt{\frac{4+4-2}{4\times 4}}\\ +1\sqrt{\frac{3+3-2}{3\times 3}}+1\sqrt{\frac{1+2-2}{1\times 2}}=23.94 \end{array}$$

The topological indices for five sulfonamide derivatives can be derived using a technique similar to that employed in Theorem 1, 2, 3,4, and Theorem 5, albeit with distinct topological indices. In Table 2, we have computed values for these indices, along with a comprehensive list of values for all medicines.

**TABLE 2:** Molecular descriptors for the candidate compounds.

Compounds	M_1_	M_2_	^m^M_2_	H	HM	F	RR	RA	S	GA	ABC
1	136	162	5.58	11.18	714	390	64.17	12.01	12.22	25.38	19.82
2	186	222	7.19	14.39	996	552	87.09	15.56	16.03	33.61	26.67
3	172	204	6.54	13.09	930	522	79.67	14.4	14.63	30.38	24.76
4	150	180	6.33	12.49	780	420	72.66	13.2	13.67	28.62	21.82
5	170	211	6.35	12.71	954	532	78.86	13.98	14.21	29.5	23.94

All degree-based topological indices were used to calculate several linear models using Eq. 1, as listed below.

#### 2.1.1 Models of regression for first zagreb index M_1_(G)

Melting Point = 401.739979445015–1.45786228160329 [M_1_(G)]

Formula Weight = 327.945883864337 + 0.185578108941419 [M_1_(G)]

#### 2.1.2 Models of regression for first zagreb index M_2_(G)

Melting Point = 392.229215752272–1.16358128576237 [M_2_(G)]

Formula Weight = 368.553655334904–0.053093234601144 [M_2_(G)]

#### 2.1.3 Models of regression for second zagreb index ^m^M_2_(G)

Melting Point = 395.746667875601–36.1592166107535 [^m^M_2_(G)]

Formula Weight = 283.74487245746 + 11.6306857678243 [^m^M_2_(G)]

#### 2.1.4 Models of regression for harmonic index H(G)

Melting Point = 412.460456296563–19.4222092308615 [H(G)]

Formula Weight = 279.663069658677 + 6.14586050276565 [H(G)]

#### 2.1.5 Models of regression for hyper zagreb index HM(G)

Melting Point = 381.262784438463–0.247899845037109 [HM(G)]

Formula Weight = 361.338523366773–0.00363571486828149 [HM(G)]

#### 2.1.6 Models of regression for forgotten index F(G)

Melting Point = 369.886126342673–0.425261023060167 [F(G)]

Formula Weight = 357.286639375047 + 0.00180331255164138 [F(G)]

#### 2.1.7 Models of regression for reciporcal randic index RR(G)

Melting Point = 398.050582636146–3.05465528351609 [RR(G)]

Formula Weight = 327.13593281809 + 0.405570233781015 [RR(G)]

#### 2.1.8 Models of regression for randic index RA(G)

Melting Point = 438.484955174762–19.8181457103949 [RA(G)]

Formula Weight = 289.559269575579 + 4.96013958238766 [RA(G)]

#### 2.1.9 Models of regression for sum connectivity S(G)

Melting Point = 412.040944213121–17.4986534915999 [S(G)]

Formula Weight = 291.494041389597 + 4.71056801938973 [S(G)]

#### 2.1.10 Models of regression for geometric arithmetic index GA(G)

Melting Point = 395.647832235126–7.83944105482153 [GA(G)]

Formula Weight = 292.301160109422 + 2.2325866123323 [GA(G)]

#### 2.1.11 Models of regression for ABC index ABC(G)

Melting Point = 412.683250150638–10.6094885116929 [ABC(G)]

Formula Weight = 303.014847528394 + 2.35634358053184 [ABC(G)]

**FIGURE 3:**
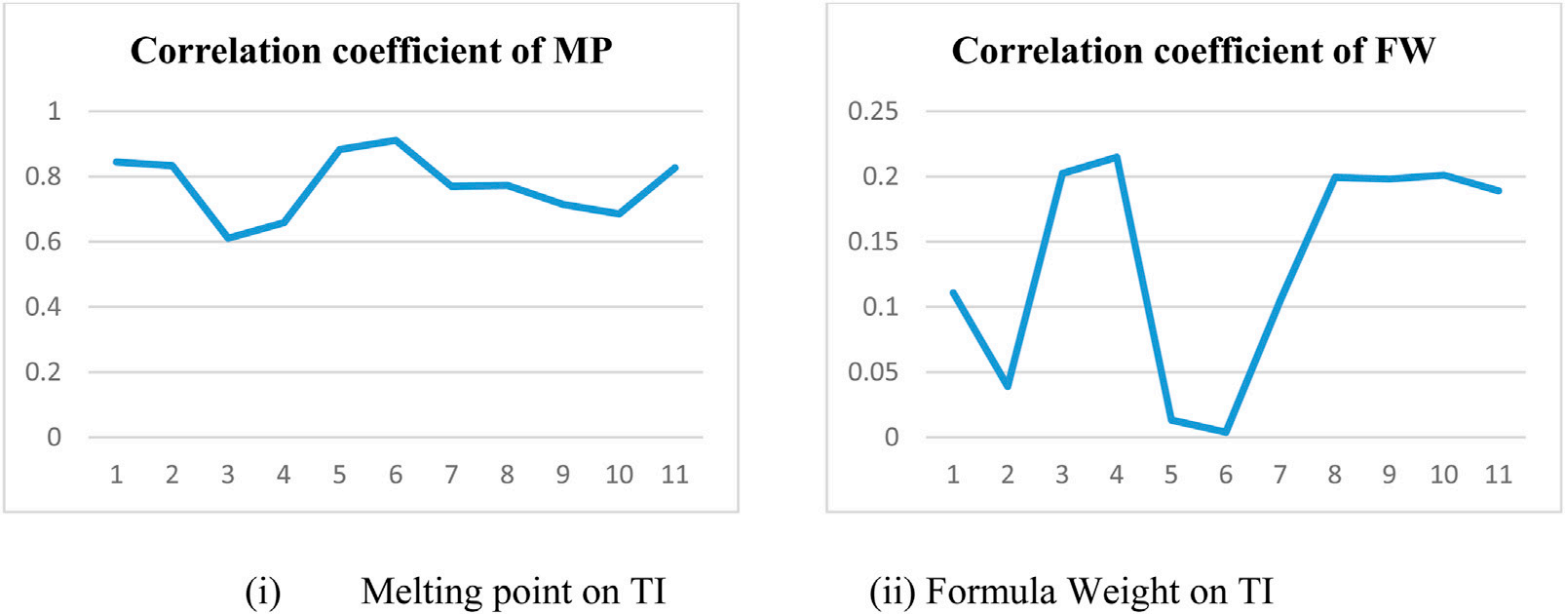
Physicochemical properties and TIs.

In a quantitative structure analysis, the comparison of topological indices and correlation coefficients of physicochemical parameters is essential. Table 3 provides the physicochemical characteristics of cancer medications, while Table 1 displays the calculated TI values derived from their molecular structures. The association correlation coefficients between TIs and two physicochemical characteristics are enumerated in Table 4. Figure 3 depicts the correlation among the topological index (TIs) and the physical and chemical characteristics of compounds, including their corresponding correlation coefficients.

**TABLE 3:** The physical characteristics of compounds.

Compounds	Melting points °C	Formula weight
4-[(2,4-dichlorobenzenesulfonamido)methyl]cyclohexane carboxylic acid	191	366.25
ethyl 4-[(2-naphthalenesulfonamido)methyl]cyclohexane carboxylate	140	375.47
ethyl 4-[(2,4-dichlorobenzenesulfonamido)methyl]cyclohexane carboxylate	133	394.3
4-[(naphthalene-2-sulfonamido)methyl]cyclohexane-1-carboxylic acid	210	347.42
(2S)-3-methyl-2-(naphthalene-1-sulfonamido)-butanoic acid	148	307.35

**TABLE 4:** Correlation coefficients (Cc).

TIs	Cc of melting point	Cc of formula weight
M_1_	0.844704869	0.110787949
M_2_	0.833038615	0.039163804
^m^M_2_	0.610871397	0.202447948
H	0.658892302	0.214820529
HM	0.883144781	0.013345128
F	0.911434835	0.003982157
RR	0.769851353	0.105314464
RA	0.772711439	0.199262466
S	0.714149035	0.198077333
GA	0.685097725	0.201026366
ABC	0.826573855	0.189148591

### 2.2 Calculation of statistical metrics/parameters

In our study, quantitative structure-property relationship (QSPR) modeling is conducted to establish a correlation between the physical and chemical properties of cancer compounds as well as their determined topological indices (TIs) of degree-based, the model of regression incorporates topological features as the independent variable. In this model, “B” stands for the constant of model, “r” indicates the correlation coefficient, and “N” signifies the number of sample compounds.

The theoretical and experimental computations highlighted in the tables emphasize a significant correlation coefficient. This testing methodology proves useful for comparisons between various models and evaluating their comparative enhancements. It is noteworthy that, in the majority of cases, the *p*-value exceeds 0.05, and the value of r surpasses 0.6. Hence, these findings suggest the significance of all attributes.

Tables 5–15 present the statistical parameters, with the abbreviations PP used for physiochemical properties, MP for melting point, and FW for formula weight.

**TABLE 5:** The utilization of statistical parameters in QSPR model for M_1_(G).

PP	N	A	B	r	*r* ^2^	F	p	Indicator
MP	5	401.74	−1.4579	0.8447	0.7135	7.4722	0.0717	Non-Significant
FW	5	327.95	0.18558	0.1108	0.0123	0.0373	0.8592	Non-Significant

**TABLE 6:** The utilization of statistical parameters in the QSPR model for M_2_(G).

PP	N	A	B	R	*r* ^2^	F	p	Indicator
MP	5	392.23	−1.1636	0.833	0.694	6.8024	0.0798	Non-Significant
FW	5	368.55	−0.0531	0.0392	0.0015	0.0046	0.9501	Non-Significant

**TABLE 7:** The utilization of statistical parameters in the QSPR model for ^m^M_2_(G).

PP	N	A	B	r	*r* ^2^	F	p	Indicator
MP	5	395.75	−36.159	0.6109	0.3732	1.7859	0.2737	Non-Significant
FW	5	283.74	11.6307	0.2024	0.041	0.1282	0.744	Non-Significant

**TABLE 8:** The utilization of statistical parameters in the QSPR model for H(G).

PP	N	A	B	r	*r* ^2^	F	p	Indicator
MP	5	412.46	−19.422	0.6589	0.4341	2.3017	0.2265	Non-Significant
FW	5	279.66	6.14586	0.2148	0.0461	0.1451	0.7286	Non-Significant

**TABLE 9:** The utilization of statistical parameters in the QSPR model for HM(G).

PP	N	A	B	r	*r* ^2^	F	p	Indicator
MP	5	381.26	−0.2479	0.8831	0.7799	10.633	0.0471	Significant
FW	5	361.34	−0.0036	0.0133	0.0002	0.0005	0.983	Non-Significant

**TABLE 10:** The utilization of statistical parameters in the QSPR model for F(G).

PP	N	A	B	r	*r* ^2^	F	p	Indicator
MP	5	369.89	−0.4253	0.9114	0.8307	14.721	0.0312	Significant
FW	5	357.29	0.0018	0.004	0.00001	0.00005	0.9949	Non-Significant

**TABLE 11:** The utilization of statistical parameters in the QSPR model for RR(G).

PP	N	A	B	r	*r* ^2^	F	p	Indicator
MP	5	398.05	−3.0547	0.7699	0.5927	4.3651	0.1279	Non-Significant
FW	5	327.14	0.40557	0.1053	0.0111	0.0336	0.8662	Non-Significant

**TABLE 12:** The utilization of statistical parameters in the QSPR model for RA(G).

PP	N	A	B	r	*r* ^2^	F	p	Indicator
MP	5	438.48	−19.818	0.7727	0.5971	4.4457	0.1255	Non-Significant
FW	5	289.56	4.96014	0.1993	0.0397	0.124	0.748	Non-Significant

**TABLE 13:** The utilization of statistical parameters in the QSPR model for S(G).

PP	N	A	B	r	*r* ^2^	F	p	Indicator
MP	5	412.04	−17.499	0.7141	0.51	3.1226	0.1754	Non-Significant
FW	5	291.49	4.71057	0.1981	0.0392	0.1225	0.7495	Non-Significant

**TABLE 14:** The utilization of statistical parameters in the QSPR model for GA(G).

PP	N	A	B	r	*r* ^2^	F	p	Indicator
MP	5	395.65	−7.8394	0.6851	0.4694	2.6535	0.2018	Non-Significant
FW	5	292.3	2.23259	0.201	0.0404	0.1263	0.7458	Non-Significant

**TABLE 15:** The utilization of statistical parameters in the QSPR model for ABC(G).

PP	N	A	B	r	*r* ^2^	F	p	Indicator
MP	5	412.68	−10.609	0.8266	0.6832	6.4704	0.0844	Non-Significant
FW	5	303.01	2.35634	0.1891	0.0358	0.1113	0.7606	Non-Significant

### 2.3 Standard error of approximation (SE) and resemblance

Standard error **(**S.E), as presented in below table (Table 16), serves as an indicator of the extent to which an analysis deviates from the approximated regression line. It also offers insights into the accuracy of predictions derived from the regression line. For additional comparisons, both practically and in theory determined predictions of the models, focusing on their physical and chemical characteristics, are included in Tables 17,18.

**TABLE 16:** Standard error of the approximation.

TIs	S.E for melting point	S.E for formula weight
M_1_	21.04309678	37.92348648
M_2_	21.75009269	38.12911197
^m^M_2_	31.12750408	37.36824216
H	29.5748222	37.26752374
HM	18.44306935	38.15498892
F	16.17627428	38.15808438
RR	25.09226673	37.94618697
RA	24.95600704	37.39316461
S	27.52082234	37.40233175
GA	28.63965167	37.37941517
ABC	22.12805235	37.46956991

**TABLE 17:** Comparison of actual and computed values for melting point from regression models.

C	MP	MP for M_1_	MP for M_2_	MP for ^m^M_2_	MP for H	MP for HM	MP for F	MP for RR	MP for RA	MP for S	MP for GA	MP for ABC
1	191	203.47	203.73	193.98	195.32	204.3	204.03	202.03	200.47	198.21	196.68	202.4
2	140	130.58	133.91	135.76	132.97	134.4	135.14	132.02	130.11	131.54	132.16	129.73
3	133	150.99	154.86	159.27	158.22	150.7	147.9	154.69	153.1	156.04	157.49	149.99
4	210	183.06	182.78	166.86	169.88	187.9	191.28	176.1	176.89	172.83	171.28	181.18
5	148	153.9	146.71	166.14	165.6	144.8	143.65	157.16	161.43	159.89	164.38	158.69

**TABLE 18:** Comparison of actual and computed values for formula weight from regression models.

C	FW	FW for M_1_	FW for M_2_	FW for ^m^M_2_	FW for H	FW for HM	FW for F	FW for RR	FW for RA	FW for S	FW for GA	FW for ABC
1	366.3	353.18	359.95	348.64	348.37	358.7	357.99	353.16	349.13	349.06	348.96	349.72
2	375.5	362.46	356.77	367.37	368.1	357.7	358.28	362.46	366.74	367	367.34	365.86
3	394.3	359.87	357.72	359.81	360.11	358	358.23	359.45	360.99	360.41	360.13	361.36
4	347.4	355.78	359	357.37	356.42	358.5	358.04	356.6	355.03	355.89	356.2	354.43
5	307.4	359.49	357.35	357.6	357.78	357.9	358.25	359.12	358.9	359.37	358.16	359.43

## 3 Conclusion

The utilization of topological indices (TIs) and statistical parameters in direct quantitative structure-property relationship (QSPR) models has revealed robust correlation coefficients across various physicochemical characteristics of medications employed for addressing cancer. The outcomes of this analysis provide beneficial perspectives for the therapeutic industry, offering guidance within creating novel treatments and establishing safety precautions for cancer therapies. The noteworthy influence of correlation coefficients between diverse topological indices (TIs) for these medications underscores the capable to predict the physical and chemical characteristics of recently identified anticancer sulfonamides compounds, especially for addressing specific cancer conditions. These results hold promise for analysts engaged in pharmaceutical research, providing a potent tool for compound discovery and development.

In particular, our analysis revealed the greatest correlation value of *r* = .911 for forgotten [F(G)] index with melting point, signifying its relevance. Additionally, the harmonic [H(G)] index demonstrated a substantial correlation of *r* = .21 with formula weight, further contributing to the understanding of these medications’ characteristics. This work not only contributes to our understanding of medications for cancer treatment but also offers practical implications for advancing pharmaceutical research and development in this critical area. In the near future, we aim to calculate the resistance distance based topological indices for the certain drugs.

## Data Availability

The original contributions presented in the study are included in the article/supplementary material, further inquiries can be directed to the corresponding author.

## References

[B1] Arockiaraj M. Celin Fiona J. Abraham J. Klavžar S. Balasubramanian K. (2024c). Guanidinium and hydrogen carbonate rosette layers: distance and degree topological indices, Szeged-type indices, entropies, and NMR spectral patterns. Heliyon 10 (3), e24814. 10.1016/j.heliyon.2024.e24814 PMC11637096 39668855

[B2] Arockiaraj M. Fiona J. C. Shalini A. J. (2024b). Comparative study of entropies in silicate and oxide frameworks. Silicon, 1–12. 10.1007/s12633-024-02892-2

[B3] Arockiaraj M. Raza Z. Maaran A. Abraham J. Balasubramanian K. (2024a). Comparative analysis of scaled entropies and topological properties of triphenylene-based metal and covalent organic frameworks. Chem. Pap., 1–24. 10.1007/s11696-023-03295-0

[B4] Aslam A. Bashir Y. Ahmad S. Gao W. (2017). On topological indices of certain dendrimer structures. Z. für Naturforsch. A 72 (6), 559–566. 10.1515/zna-2017-0081

[B5] Boufas W. Dupont N. Berredjem M. Berrezag K. Becheker I. Berredjem H. (2014). Synthesis and antibacterial activity of sulfonamides. SAR and DFT studies. J. Mol. Struct. 1074, 180–185. 10.1016/j.molstruc.2014.05.066

[B6] Burger A. Abraham D. J. (2003) Burger's medicinal chemistry and drug discovery. No Title).

[B7] Cheng X.-C. Wang Q. Fang H. Xu W. F. (2008). Role of sulfonamide group in matrix metalloproteinase inhibitors. Curr. Med. Chem. 15 (4), 368–373. 10.2174/092986708783497300 18288991

[B8] Chidambaram N. Kamran M. Balasubramanian D. Hameed S. Mehmood S. Raza W. (2024). Novel distance-based molecular descriptors for styrene butadiene rubber structures. JTAM J. Teori Dan. Apl. Mat. 8 (2), 411–424. 10.31764/jtam.v8i2.20037

[B9] Crespo R. de Bravo M. G. Colinas P. A. Bravo R. D. (2010). *In vitro* antitumor activity of N-glycosyl sulfonamides. Bioorg. Med. Chem. Lett. 20 (22), 6469–6471. 10.1016/j.bmcl.2010.09.052 20888767

[B10] Danish M. Akhtar A. Ashfaq M. Arshad M. N. Asiri A. M. (2021). Synthesis crystal structure and spectral properties of new sulfonamides. J. Chem. Crystallogr. 51, 543–552. 10.1007/s10870-021-00878-1

[B11] Danish M. Bibi A. Gilani K. Raza M. A. Ashfaq M. Arshad M. N. (2019). Antiradical, antimicrobial and enzyme inhibition evaluation of sulfonamide derived esters; synthesis, X-Ray analysis and DFT studies. J. Mol. Struct. 1175, 379–388. 10.1016/j.molstruc.2018.07.116

[B12] Danish M. Tahir M. N. Hussain A. Ashfaq M. Sadiq M. N. (2015a). Crystal structure of 4-{[(naphthalen-2-yl) sulfonylamino] methyl} cyclohexanecarboxylic acid. Acta Crystallogr. Sect. E Crystallogr. Commun. 71 (3), o145. 10.1107/s2056989015002054 25844218 PMC4350692

[B13] Danish M. Tahir M. N. Jabeen N. Raza M. A. (2015b). Crystal structure of (2S)-3-methyl-2-[(naphthalen-1-ylsulfonyl) amino] butanoic acid. Acta Crystallogr. Sect. E Crystallogr. Commun. 71 (5), o308. 10.1107/s2056989015007057 25995919 PMC4420138

[B14] Das B. Reddy V. S. Reddy M. R. (2004). An efficient and selective tosylation of alcohols with p-toluenesulfonic acid. Tetrahedron Lett. 45 (36), 6717–6719. 10.1016/j.tetlet.2004.07.076

[B15] Das K. C. Gutman I. Furtula B. (2011). On atom-bond connectivity index. Chem. Phys. Lett. 511 (4-6), 452–454. 10.1016/j.cplett.2011.06.049

[B16] Duchowicz P. R. Talevi A. Bruno-Blanch L. E. Castro E. A. (2008). New QSPR study for the prediction of aqueous solubility of drug-like compounds. Bioorg. Med. Chem. 16 (17), 7944–7955. 10.1016/j.bmc.2008.07.067 18701302

[B17] Fajtlowicz S. (1988). “On conjectures of Graffiti,” in Annals of discrete mathematics (Elsevier), 113–118.

[B18] Farahani M. R. (2013). On the Randic and sum-connectivity index of nanotubes. Ann. West Univ. Timisoara-Mathematics Comput. Sci. 51 (2), 39–46. 10.2478/awutm-2013-0014

[B19] Furtula B. Gutman I. (2015). A forgotten topological index. J. Math. Chem. 53 (4), 1184–1190. 10.1007/s10910-015-0480-z

[B20] Groutas W. C. He S. Kuang R. Ruan S. Tu J. Chan H. K. (2001). Inhibition of serine proteases by functionalized sulfonamides coupled to the 1, 2, 5-thiadiazolidin-3-one 1, 1 dioxide scaffold. Bioorg. Med. Chem. 9 (6), 1543–1548. 10.1016/s0968-0896(01)00037-2 11408173

[B21] Gutman I. Furtula B. Elphick C. (2014) “Three new/old vertex-degree-based topological indices,” in MATCH communications in mathematical and in computer chemistry.

[B22] Gutman I. Furtula B. Katanić V. (2018). Randić index and information. AKCE Int. J. Graphs Comb. 15 (3), 307–312. 10.1016/j.akcej.2017.09.006

[B23] Gutman I. Polansky O. E. (2012) Mathematical concepts in organic chemistry. Springer Science and Business Media.

[B24] Gutman I. Trinajstić N. (1972). Graph theory and molecular orbitals. Total φ-electron energy of alternant hydrocarbons. Chem. Phys. Lett. 17 (4), 535–538. 10.1016/0009-2614(72)85099-1

[B25] Hansen P. J. Jurs P. C. (1988). Chemical applications of graph theory. Part I. Fundamentals and topological indices. J. Chem. Educ. 65 (7), 574. 10.1021/ed065p574

[B26] Hayat S. Arshad M. Khan A. (2024b). Graphs with given connectivity and their minimum Sombor index having applications to QSPR studies of monocarboxylic acids. Heliyon 10 (1), e23392. 10.1016/j.heliyon.2023.e23392 38163160 PMC10755296

[B27] Hayat S. Khan A. Ali K. Liu J. B. (2024a). Structure-property modeling for thermodynamic properties of benzenoid hydrocarbons by temperature-based topological indices. Ain Shams Eng. J. 15 (3), 102586. 10.1016/j.asej.2023.102586

[B28] Hosamani S. Perigidad D. Jamagoud S. Maled Y. Gavade S. (2017). QSPR analysis of certain degree based topological indices. J. Statistics Appl. Probab. 6 (2), 361–371. 10.18576/jsap/060211

[B29] Markgren P.-O. Schaal W. Hämäläinen M. Karlén A. Hallberg A. Samuelsson B. (2002). Relationships between structure and interaction kinetics for HIV-1 protease inhibitors. J. Med. Chem. 45 (25), 5430–5439. 10.1021/jm0208370 12459011

[B30] Mohammed M. A. Atan K. A. Khalaf A. M. Said M. R. Hasni R. (2016). “On atom bond connectivity index of some molecular graphs,” in AIP conference proceedings (AIP Publishing), 1739, No. 1.

[B31] Natarajan C. Selvamuthukumaran S. Farahani M. R. (2022) On leap Zagreb indices of some cycle related graphs.

[B32] Penning T. D. Talley J. J. Bertenshaw S. R. Carter J. S. Collins P. W. Docter S. (1997). Synthesis and biological evaluation of the 1, 5-diarylpyrazole class of cyclooxygenase-2 inhibitors: identification of 4-[5-(4-methylphenyl)-3-(trifluoromethyl)-1 H-pyrazol-1-yl] benzenesulfonamide (SC-58635, celecoxib). J. Med. Chem. 40 (9), 1347–1365. 10.1021/jm960803q 9135032

[B33] Ramírez Alfaro J. J. (2022) Estudio de La concentración de metales en el proceso de una empresa productora de premezclas alimenticias granuladas.

[B34] Rotella D. P. (2002). Phosphodiesterase 5 inhibitors: current status and potential applications. Nat. Rev. Drug Discov. 1 (9), 674–682. 10.1038/nrd893 12209148

[B35] Shanmukha M. Usha A. Praveen B. M. Douhadji A. (2022). Degree-based molecular descriptors and QSPR analysis of breast cancer drugs. J. Math. 2022, 1–13. 10.1155/2022/5880011

[B36] Shirdel G. Rezapour H. Sayadi A. (2013) The hyper-Zagreb index of graph operations.

[B37] Supuran C. T. Scozzafava A. (2002). Applications of carbonic anhydrase inhibitors and activators in therapy. Expert Opin. Ther. Pat. 12 (2), 217–242. 10.1517/13543776.12.2.217

[B38] Talley J. J. Brown D. L. Carter J. S. Graneto M. J. Koboldt C. M. Masferrer J. L. (2000). 4-[5-Methyl-3-phenylisoxazol-4-yl]-benzenesulfonamide, valdecoxib: a potent and selective inhibitor of COX-2. J. Med. Chem. 43 (5), 775–777. 10.1021/jm990577v 10715145

[B39] Ullah A. Zaman S. Hamraz A. Muzammal M. (2023a). On the construction of some bioconjugate networks and their structural modeling via irregularity topological indices. Eur. Phys. J. E 46 (8), 72. 10.1140/epje/s10189-023-00333-3 37605051

[B40] Ullah A. Zaman S. Hussain A. Jabeen A. Belay M. B. (2023b). Derivation of mathematical closed form expressions for certain irregular topological indices of 2D nanotubes. Sci. Rep. 13 (1), 11187. 10.1038/s41598-023-38386-1 37433876 PMC10336147

[B41] Vukičević D. Furtula B. (2009). Topological index based on the ratios of geometrical and arithmetical means of end-vertex degrees of edges. J. Math. Chem. 46, 1369–1376. 10.1007/s10910-009-9520-x

[B42] Wiener H. (1947). Structural determination of paraffin boiling points. J. Am. Chem. Soc. 69 (1), 17–20. 10.1021/ja01193a005 20291038

[B43] Winum J. Y. Scozzafava A. Montero J. L. Supuran C. T. (2006). Therapeutic potential of sulfamides as enzyme inhibitors. Med. Res. Rev. 26 (6), 767–792. 10.1002/med.20068 16710859

[B44] Yan T. Kosar Z. Aslam A. Zaman S. Ullah A. (2023). Spectral techniques and mathematical aspects of K 4 chain graph. Phys. Scr. 98 (4), 045222. 10.1088/1402-4896/acc4f0

[B45] Zaman S. He X. (2022). Relation between the inertia indices of a complex unit gain graph and those of its underlying graph. Linear Multilinear Algebra 70 (5), 843–877. 10.1080/03081087.2020.1749224

[B46] Zaman S. Jalani M. Ullah A. Ahmad W. Saeedi G. (2023). Mathematical analysis and molecular descriptors of two novel metal–organic models with chemical applications. Sci. Rep. 13 (1), 5314. 10.1038/s41598-023-32347-4 37002273 PMC10066202

[B47] Zhong J. Gan X. Alliston K. R. Lai Z. Yu H. Groutas C. S. (2004). Potential protease inhibitors based on a functionalized cyclic sulfamide scaffold. J. Comb. Chem. 6 (4), 556–563. 10.1021/cc030047r 15244417

